# Analgesic Efficacy of Intravenous Ibuprofen in the Treatment of Postoperative Acute Pain: A Phase III Multicenter Randomized Placebo-ControlledDouble-Blind Clinical Trial

**DOI:** 10.1155/2023/7768704

**Published:** 2023-03-07

**Authors:** Hong-Su Zhou, Ting-Ting Li, Yu Pi, Ting-Hua Wang, Fei Liu, Liu-Lin Xiong

**Affiliations:** ^1^Department of Anesthesiology, Affiliated Hospital of Zunyi Medical University, Zunyi, Guizhou 563000, China; ^2^Department of Anesthesiology, West China Hospital, Sichuan University, Chengdu 610044, China; ^3^Department of Anesthesiology, South West Medical University, Luzhou 646000, China

## Abstract

**Objective:**

To evaluate the analgesic efficacy and safety of different does of intravenous ibuprofen (IVIB) in the treatment of postoperative acute pain.

**Methods:**

Patients with an intravenous (IV) patient-controlled analgesia device after abdominal or orthopedic surgery were randomly divided into placebo, IVIB 400 mg, and IVIB 800 mg groups. The first dosage of study medicines was given intravenously 30 minutes (min) before surgery ended, followed by six hours (h) intervals for a total of eight doses following surgery. The demographic characteristics and procedure data, cumulative morphine consumption, the visual analog scale (VAS), the area under the curve (AUC) of VAS, patient satisfaction score (PSS), the rates of treatment failure (RTF), and adverse events (AEs) and serious adverse event (SAEs) were recorded during the period of trial.

**Result:**

A total of 345 patients were enrolled in the full analysis set (FAS), and of 326 participants were valid data set (VDS). Demographic characteristics, disease features, and medical history of patients were not significantly different between groups. Total morphine consumption of the IVIB 400 mg group (11.14 ± 7.14 mg; *P* = 0.0011) and the IVIB 800 mg group (11.29 ± 6.45 mg; *P* = 0.0014) was significantly reduced compared with the placebo group (14.51 ± 9.19 mg) for 24 h postoperatively, there was no significant difference between the IVIB 400 mg and IVIB 800 mg groups (*P* = 0.9997). The placebo group had significantly higher VAS and the AUCs of VAS than those in the IVIB 400 mg and the IVIB 800 mg groups at rest and movement for 24 h postoperatively (*P* *<* 0.05), and there was no significant difference between the IVIB 400 mg and IVIB 800 mg groups (*P* > 0.05). RTF was slightly higher in the placebo group than IVIB 400 mg group and 800 mg group, and no statistical significance (*P* < 0.690). PSS in the IVIB 400 mg (*P* = 0.0092) and the IVIB 800 mg groups (*P* = 0.0011) was higher than the placebo group for pain management, there was also no significant difference between the IVIB 400 mg and IVIB 800 mg groups (*P* = 0.456). The incidence of RTF (*P* = 0.690) and AEs (*P* > 0.05) were not different among the three groups.

**Conclusion:**

Intermittent IV administration of ibuprofen 400 mg or 800 mg within 24 h after surgery in patients undergoing abdominal and orthopedic surgery significantly decreased morphine consumption and relieved pain, without increasing the incidence of AEs.

## 1. Introduction

Postoperative pain has long been a major concern for patients. A Spanish multicenter cross-sectional survey found that 73% of patients experienced acute postoperative pain [1]. About 66% of patients in the United States had still suffered from moderate to severe postoperative pain [2]. In one large cohort study, three-fourths of the surgeries with the highest pain scores were orthopedic or orthopedic trauma surgeries, the rest were common minor-tomedium-level surgical procedures, including some with laparoscopic approaches, appendectomy, cholecystectomy, hemorrhoidectomy, and tonsillectomy [3]. Therefore, postoperative pain seems unavoidable for most surgeries, and often lasts for several days, especially on the first postoperative day [3, 4]. Inadequate postoperative pain relief may cause an increased incidence of cardiac and pulmonary complications [5, 6], and reduce the vitality of the gastrointestinal [7], and even cause chronic postoperative pain, and affecting the quality of life for hospitals and patients [8]. In brief, severe postoperative pain remains a widespread event and postoperative pain management has always been a challenge for most clinicians.

Opioids are widely used in acute pain treatment because of their powerful analgesic effect. Early studies have shown that more than half of patients received morphine to relieve moderate or severe postoperative pain [9, 10]. However, it is difficult to completely control pain experience with a single opioid, especially for inflammatory pain. Because it blocks the transmission of nerve impulses and prevent the sensation of pain, mainly by binding to pain-related*μ*, *κ*, and *δ* receptors, which is not the most effective against pain caused by nerve injury and inflammation, and one of the reasons for the failure of postoperative acute pain management [11, 12]. In addition, opioids have many adverse effects such as nausea, dizziness, vomiting, drowsiness, pruritus, rash, urinary retention, delayed gastrointestinal motility, and even respiratory depression [13]. The American Society of Anesthesiologists (ASA) recommends [14] that a multimodal analgesic protocol contributes to managing acute perioperative pain. Multimode analgesia can not only significantly improve patients' satisfaction with postoperative analgesia, but also reduce the use of opioids, thus reducing related adverse reactions [14–16]. Clinically, opioids matching with NSAIDs are very popular for postoperative pain due to ease of operation, safety, and personalization.

There are various NSAIDs in clinical, but they may increase the risk of gastrointestinal bleeding with limited onset of action and repeated doses [17]. Ibuprofen, as one of the most commonly used pain relief medications, is generally available to patients worldwide without the need for a prescription and has both central and predominantly peripheral nervous system actions [18, 19]. By stimulating anti-inflammatory activity, it can inhibit the inflammatory cascade triggered by invasive procedures, lighten pain, and lower body temperature while avoiding its progression.

Concerning adverse events (AEs), a study confirmed that ibuprofen is less prone to gastrointestinal (GI) and cardiovascular than ketorolac and diclofenac [20], and the same conclusion was shown in a meta-analysis conclusion [21]. Additionally, it is also the only NSAID approved for pain and fever [22]. Though efficacy and safety have been demonstrated [23–26], its conclusions were ambiguous for the management of acute pain after surgery in adults in the recent review [27]. Therefore, the purpose of this study was to further evaluate the efficacy and safety for different does of IVIB in the treatment of postoperative acute pain.

## 2. Methods

### 2.1. Ethics and Registration

The ethics of the study has been approved. Drug clinical research approval number: 2014L01894; date of registration: 16/01/2015. All methods were performed by the relevant guidelines and regulations. Informed consent forms and all amendments were reviewed and approved (due to the need of blind audit, the specific audit agency is temporarily hidden).

### 2.2. Study Design

This study was a prospective, randomized, double-blind, placebo-controlled trial. This study was conducted in 10 hospitals throughout China from Jul.10, 2015, to Dec.25, 2016.

#### 2.2.1. Inclusion Criteria

**Figure 1:**
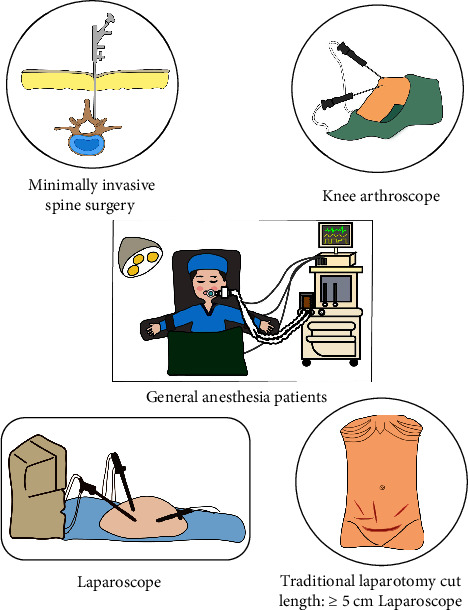
Patients, included in the trial plan, underwent general anesthesia and different types of abdominal and orthopedic surgery.

**Figure 2:**
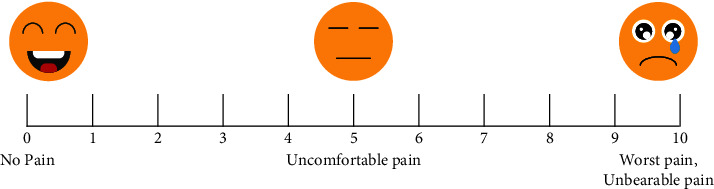
Patients were instructed to circle the number in the line above that reflects their current level of pain at rest and during movement. Min: minutes; h: hour.

A total of 360 patients under general anesthesia were included in this study, all of whom were abdominal and orthopedic surgery patients with incisions length 5 cm at least and who required postoperative hospitalization (Figure1 depicts several types of surgery). In addition, they were expected to receive patient-controlled IV analgesia (PCIA) of morphine for over 24 hours (h). Included patients were 18 and 75 years old, including males and females with the American Society of Anesthesiologists (ASA) physical statuses I-III, who were able to self-report changes in pain (supplemental instrument Table 1). Pain intensity was measured by patient self-assessment, and reflected via circling the corresponding number in Figure 2.

#### 2.2.2. Exclusion Criteria

Patients with weight <40 kg, BMI ≤ 18 kg/m^2^ and/or BMI ≥ 30 kg/m^2^, a history of asthma or heart failure, or pregnant or nursing or poorly controlled hypertension (systolic blood pressure ≥140 mmHg, or diastolic blood pressure ≥90 mmHg) were also excluded. Patients were excluded from the study if they had a history of allergy or hypersensitivity to NSAIDs or used NSAIDs within 12 h, or had a history of tolerance or dependence on narcotics or opioids. Patients with kidney dysfunction (creatinine is greater than 1.5 times the limit of the baseline, or on dialysis treatment within 28 days before surgery) or liver dysfunction (ALT or/and AST was greater than 1.5 times the limit of the baseline) were unable to participate in this study. Patients were considered when they had no underlying bleeding factors such as hemophilia, thrombocytopenia, abnormal platelet function; or no used other analgesics, muscle relaxants, or sedatives within 24 h prior to surgery; or no local anesthesia, nerve blocks, epidurals, and another analgesia preoperatively/intraoperatively. Patients were excluded if taking warfarin, lithium, combined angiotensin-converting enzyme inhibitors (ACEI), and diuretics at present; or with other events deemed by the investigator that were not eligible, for example, enrolled in other clinical studies within 3 months (supplemental instrument Table 2).

#### 2.2.3. Randomization and Group

**Figure 3:**
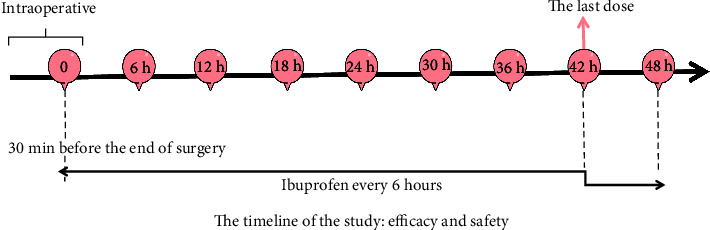
Study design.

**Figure 4:**
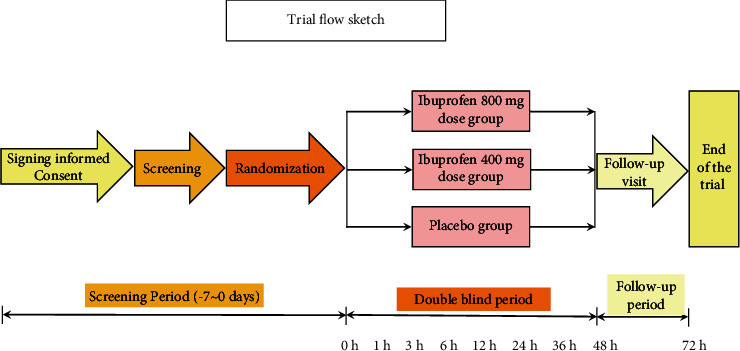
The whole clinical trial sketch.

The method of centralized random grouping was adopted, and each research center competed to be included in the group. During the first 48 h of the study period subjects were randomized in a 1 : 1 : 1 ratio to the IVIB 400 mg group (diluted to 200 ml 0.9% NaCl), the IVIB 800 mg group (diluted to 200 ml 0.9% NaCl), and the placebo group (200 ml 0.9% NaCl) using a double-blind, simple randomization protocol. The first dose of ibuprofen or placebo was administered 30 minutes before the end of the procedure. Subsequently, interventions were performed every 6 h until the end of the whole clinical trial, with a total of 8 doses (Figure 3). All patients are receiving the same IV morphine (0.5 mg/ml) patient-controlled analgesia (PCA). The background dose was 0.25 mg/h, the supplementary dose was 1 mg/5 min, and the maximum daily dose was not more than 60 mg morphine. Sufentanil (0.2-0.3 ug/kg) was used for analgesia during anesthesia induction in all cases. Anesthetic maintenance was maintained by continuous administration of sevoflurane (1.3 minimum alveolar concentration (MAC)) and remifentanil (0.1–0.2 *µ*g/kg/min, the actual dosage was adjusted appropriately through changes in the patient's vital signs). The trial medication and prophylactic antiemetics (5-HT3 receptor antagonists (e.g., granisetron and ondansetron)) was given at half an hour before the end of the operation. There were no other catheter-based regional anesthetic techniques Figure 4

#### 2.2.4. Blinding

Researchers, patients, and caregivers were unaware of the intervention allocation, and only supervising physicians were known.

#### 2.2.5. Efficacy Assessment

*(1) Primary Outcome*. Total morphine use in 24 hours after surgery.

*(2) Secondary Outcomes*. ① Visual analogue scale (VAS) was measured by patient self-assessment both at rest and with movement (at 1, 3, 6, 12, 24, 36, and 48 h following surgery). Different surgical types of postoperative pain with movement may bring on or aggravated by differently movement. In this paper, cough, physiotherapy, sitting, standing, rolling over in bed, and active flexion and extension were specifically described as pain-evoking maneuvers. ② The area under the curve (AUC) of VAS (1–24 h, 6–24 h, and 12–24 h to determine the differences in overall pain at differing time points) at rest and with movement; ③ the rates of treatment failure (RTF), defined as the rate of analgesia with other nonmorphine drugs within 24 h after the end of surgery; ④ patient satisfaction score (assessed by a 5-point Likert scale: excellent-5;good-4;moderate-3;pass-2; and no pass-1) was recorded on postoperative day 2.

#### 2.2.6. Safety Assessment

Specific AEs associated with IVIB include renal dysfunction, cardiovascular events, GI or operative site bleeding, thrombophlebitis, gastrointestinal dysfunction (nausea, flatulence, and vomiting), and nervous system (headache and dizziness). Opioid-related specific AEs include nausea, vomiting, pruritus, respiratory depression, sedation, urinary retention, and allergic reactions/rash [28].

### 2.3. Statistical Analysis

#### 2.3.1. Sample Size

To hypothesis that the effect of the 800 mg ibuprofen group was better than the placebo group, and the study design was 1 : 1, when the main efficacy point was the total dose of morphine 24 h after surgery. The parameters were set: bilateral *α* = 0.025, 1-*β* = 80%. The data showed a difference of 8.6 mg in total dosage of morphine between the IVIB 800 mg ibuprofen group and the placebo group 24 h after surgery, with a combined variance of 400 mg [29]. According to the sample size formula (calculated by PASS software) for the comparison of two means, a total of 105 cases were required per group. Considering 15% shedding during the clinical trials, we expect each group to shed an additional 15 cases, which is calculated by multiplying 105 by 15%. The total samples for each group (the placebo, the IVIB 400 mg, and the IVIB 800 mg group) are 105 plus 5, which is 120. That is, the total samples for the three groups are 360, and was randomized equally to the placebo, the IVIB 400 mg, and the IVIB 800 mg group.

Percentages present categorical variables. Interquartile range presents level variable. If the continuous variables conformed to a normal distribution, parametric tests, such as one-way analysis of variance (ANOVA), were used, and the results were expressed as means ± standard deviation (SD); if they did not conform to a normal distribution, nonparametric tests, such as rank sum tests, were used, and the results were expressed as median and interquartile range. Therefore, ANOVA and the LSD test were used to compare different groups' continuous variables (VAS scores, the AUC of VAS, and the cumulative morphine dose). Laboratory assessments were analyzed using paired *t*-tests for the data before and after treatment within each group, if the variable followed normal distribution. AEs, SAEs, and RTF were tested by the *χ*^2^ test or fisher's exact to compare differences between groups. Medical history and PSS was analyzed using a Kruskal–Wallis H test. *P* < 0.05 was considered statistically significant (two-sided test). All statistics were analyzed in SAS 9.4 software (SAS Institute Inc.).

## 3. Results

**Figure 5:**
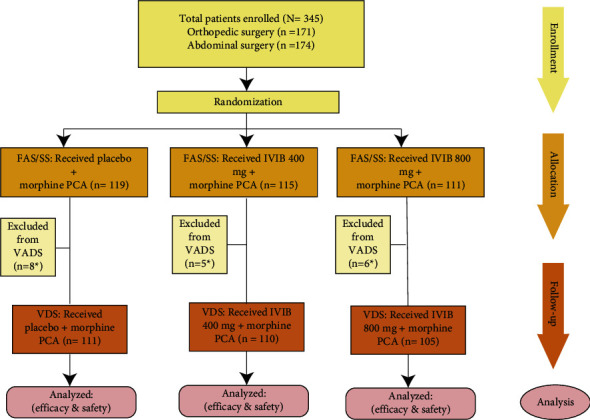
Distribution of surgery patients randomized to receive IVIB or placebo for postoperative pain. PCA, patient-controlled analgesia. FAS, full analysis set including all patients who were randomized and received at least a partial dose of IV ibuprofen or placebo; VAD, valid data set including all patients who receive at least 4 doses of IV ibuprofen or placebo as required by the study protocol; SS, safety set, including all patients who received at least a dose of IV ibuprofen or placebo and safety assessment after randomization. ^*∗*^Reasons for exclusion from the efficacy population included adverse events, noncompliance, lack of efficacy, and withdrawn consent.

A total of 345 patients were enrolled to full analysis set (FAS, including all patients who were randomized and received at least a partial dose of IV ibuprofen or placebo). Of these, 326 populations were enrolled to valid data set (VDS, including all patients who receive at least 4 doses of IV ibuprofen or placebo as required by the study protocol) (Figure 5). Baseline demographic characteristics were not significantly different between the three groups (Table 1).

### 3.1. Efficacy Assessment

#### 3.1.1. Primary Efficacy Variable

**Figure 6:**
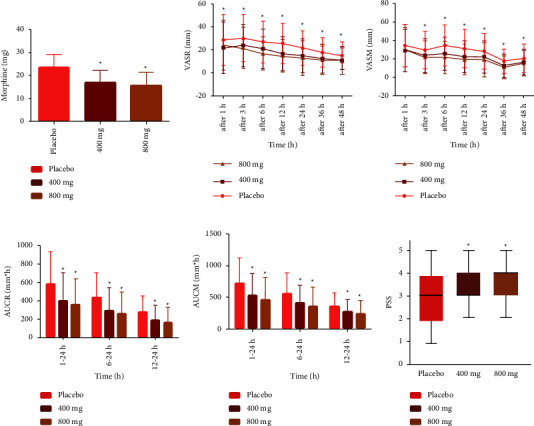
The efficacy assessment of IVIB during 48 h postoperatively. (a) Morphine dose during the 24 h postoperative administration. (b) VAS at rest. (c) VAS at movement. (d) The AUCs of VAS at rest (mm*∗*h). (e) The AUCs of VAS at movement (mm*∗*h). (f) PSS for pain treatment during hospitalization (PSS was assessed by a 5-point Likert scale: excellent-5;good-4;moderate-3;pass-2; and no pass-1; the bar represents satisfaction score, the bold bar represents the median, the upper and lower limit represent the highest and lowest score, respectively) ^*∗*^*P* < 0.05, vs. placebo (including IVIB 400 mg vs placebo; IVIB 800 mg vs placebo). VAS, visual analog scale; VASR, visual analog scale at rest; VASM, visual analog scale with movement. AUCR, the area under the curve of VAS at rest; AUCM, the area under the curve of VAS at movement; PSS, patient satisfaction scores; h, hour.

IVIB 400 mg group (11.14 ± 7.14 mg; *P* = 0.0011) and the IVIB 800 mg group (11.29 ± 6.45 mg; *P* = 0.0014) morphine consumption was significantly reduced compared with the placebo group (14.51 ± 9.19 mg, Figure 6(a)) in postoperative 24 h. The morphine use between the IVIB 400 mg group and the IVIB 800 mg group was not significantly different (*P* = 0.9997).

#### 3.1.2. Secondary Efficacy Variables

Compared with the placebo group, the VAS evaluation at rest, regardless of the IVIB 400 mg or IVIB 800 mg groups (*P*_1_ represents the placebo group VS IVIB 400 group; *P*_2_ represents the placebo group VS the IVIB 800 group), was significantly reduced at 1 h (*P*_1_ = 0.0097; *P*_2_ = 0.0105), 3 h (*P*_1_ = 0.0264; *P*_2_ = 0.0034), 6 h (*P*_1_ = 0.0066; *P*_2_ < 0.0001), 12 h (*P*_1_ < 0.0001; *P*_2_ < 0.0001), 24 h ((*P*_1_ = 0.0001; *P*_2_ < 0.0001), 36 h (*P*_1_ = 0.0005; *P*_2_ < 0.0001), and 48 h (*P*_1_ = 0.0030; *P*_2_ = 0.0006) after surgery over time (Figure 6(b)); for the VAS evaluation with movement, IVIB 400 mg and IVIB 800 mg were also significantly reduced at 3 h (*P*_1_ = 0.0264; *P*_2_ = 0.0034), 6 h (*P*_1_ = 0.0026; *P*_2_ *<* 0.0001), 12 h (*P*_1_ = 0.0013; *P*_2_ *<* 0.0001), 24 h (*P*_1_ = 0.0122; *P*_2_ = 0.0001), 36 h (*P*_1_ = 0.0005; *P*_2_ < 0.0001), 48 h (*P*_1_ = 0.0302; *P*_2_ = 0.0096) after surgery, compared with the placebo group (Figure 6(c)). Parallelly, the IVIB 400 mg and 800 mg groups scored significantly lower than that placebo group across all 3 time periods for the AUCs of VAS at rest or movement (All *P* < 0.0001) (Figures 6(d) and 6(e)). There were no difference between the IVIB 400 mg group and the IVIB 800 mg group in VAS at rest and during movement, and the AUCs of VAS in any study period (supplemental instrument Tables S3–S6). RTF was slightly higher in the placebo group (17.65%) than the IVIB 400 mg (15.65%) group and the 800 mg group (13.51%), and no statistical significance (*P* = 0.6984). PSS was higher in the IVIB 400 mg (*P* = 0.0092) and the IVIB 800 mg (*P* = 0.0011) group than in the placebo group, but not significantly different from the IVIB 400 mg and IVIB 800 mg groups (*P* = 0.456, Figure 6(f)).

#### 3.1.3. Safety Analysis

Safety was evaluated by AEs, vital signs, and laboratory assessments of patients. AEs and SAEs were reported in the placebo group of 119 patients (58.26% and 0), IVIB 400 mg group of 115 patients (60.36% and 1.74%), and IVIB 800 mg group of 111 patients (68.91% and 2.70%), respectively, and there were no differences between groups (*P*_AEs_ = 0.2015 and *P*_SAEs_ = 0.1695). Supplemental instrument Table S7 listed the safety assessments of the three groups over the course of the study. Of these, Table 2 shows AEs associated with ibuprofen and morphine, and illustrates the statistical results, which were not significantly different for the three groups. SAEs, such as intestinal fistula, acute cholecystitis, and abdominal infection, were evaluated for correlation with the adverse effects of the study drugs and were mostly unrelated (Table 3). Meanwhile, we also found that the incidence of pyrexia was higher in the placebo group compared to the IVIB 400 mg and 800 mg (*P* = 0.0002). Furthermore, the placebo group had a significantly higher body temperature than the IVIB group, from 6 h after the administration of the first dose to the end of the study (All *P* < 0.05, Supplemental instrument Table S8). Other vital signs (pulse, respiration, and blood pressure), blood routine, biochemistry, and coagulation were not different between groups (Tables S9–S11). No deaths were reported during the study period.

## 4. Discussion

This study evaluated the analgesic efficacy and the safety of IV ibuprofen-assisted postoperative pain in patients undergoing abdominal and orthopedic surgery. The results of this study showed that IVIB 400 mg and 800 mg not only reduced morphine consumption, compared with the placebo group, but also provided a significant reduction in pain scores at rest and with movement, both VAS and the AUCs of VAS on postoperative days 1 and 2 on the ward. Besides, the recognized AEs associated with opioid analgesia and ibuprofen did not increase significantly for the incidence of AEs between groups.

### 4.1. Efficacy

In the present study, a meaningful result was that intermittent intravenous administration of ibuprofen starting 30 min before the end of surgery significantly narrowed morphine consumption compared with placebo, also reduced patients' postoperative VAS and the AUCs of VAS both at rest and movement. What's more, patients treated with dose of IVIB 400 mg or 800 mg, earned statistically higher satisfaction than placebo group, and lowered the body temperature. Early clinical trials have demonstrated that IVIB administered over 30–60 minutes is highly effective in treating pain and even fever [29], and that accelerating the speed of IV administration shortens the onset of action, which has a half-life of 2 h in most adults and blood concentration is about twice that of oral ibuprofen [30]. Typical IV doses for analgesia in adults are 400 mg to 800 mg every 6 h as needed up to a maximum dose of 3200 mg per day [31].

The findings of the present study were that IVIB may provide effective analgesia, regardless of 400 mg or 800 mg, when administering pre-emptively during the perioperative period. Interestingly, we found that the dose was different in previous studies of single-dose treatment of postoperative pain. Homologous conclusions are supported by two systematic reviews and meta-analyzes published in recent years [12, 32]. Karaca concluded that IVIB 400 mg was definitely beneficial for postoperative pain of laparoscopic surgery compared to placebo [33], which is consistent with our result. Distinctively, Liu and his colleagues preferred to recommend IVIB 800 mg, because total morphine consumption was significantly reduced in the ibuprofen 800 mg group versus the placebo group, but, was not significantly different between the placebo and ibuprofen 400 mg groups [34]. Convincingly, the same treatment for postoperative pain might have a completely different effect because of patient characteristics, type of surgery, and anesthetic technique, which may contribute to identify factors associated with optimal multimodal analgesia for pain management. So far, the vast majority of postoperative pain studies focus on IVIB 800 mg. A narrative summary described that 344 adult patients and healthy volunteers were included in these 9 studies, 200 of these subjects received IVIB, and the remaining 144 received either placebo or a comparator medication, suggesting that the patients with IVIB 800 mg experienced less postoperative pain, decreased opioid use, improved quality of recovery, and even used less over-the-counter medication [12]. Recently, the IVIB 800 mg infusion was utilized for the management of postoperative pain after percutaneous nephrolithotomy, gynecologic laparoscopy, and orthognathic surgery, which suggested that it had beneficial effect in pain control, and even provided effective preventive analgesia when administered 30 min before surgery [35–37]. As well, satisfaction with pain management was documented in different clinical trials, and confirmed that satisfaction was significantly higher with both doses than with placebo [34, 36]. Arguably, ibuprofen plays a very beneficial role in urgent postoperative pain relief, which is non-negligible to optimize patient comfort and improving patient prognosis. What's more, IVIB also could provide a more robust fever reduction in those patients with a postoperative high fever, which was related to decrease levels of catecholamines, cortisol, and cytokines, and suppressed inflammatory responses [23]. Parallelly, there was evidence that febrile patients receiving rapid IVIB 400 mg and 800 mg could reduce body temperature and undoubtedly fever-related symptoms and side effects [38]. In short, besides gaining higher patient satisfaction and reducing the risk of fever, different doses of IVIB provided further evidence of the effectiveness of multimodal analgesic protocols in the management of acute postoperative pain. Therefore, we believed that IVIB 400 mg and 800 mg were tryable for postoperative pain management abdominal and orthopedic surgery.

### 4.2. Safety

Although IVIB may avoid sensitization of pain receptors by inhibiting the inflammatory cascade response resulting from invasive procedures, there is some safety issues associated with the use of NSAIDs. In the data of this study, GI bleeding and renal events, regarded as acute safety profile, occurred in only one case in the IVIB 400 mg group. Importantly, the occurrence of common AEs, including naupathia, vomiting, constipation, headache, flatulence, pruritus, and dizziness, caused almost no meaningful safety problems, compared to placebo. There was evidence of an elevated risk when IVIB is infused for more than 5 days, or a high-risk group, owing in part to its selectivity for the cyclooxygenase-1 (COX1) enzyme [39]. A recent meta-analysis concerning to different fixed-dose combination (FDC) of ibuprofen plus paracetamol concluded that 400 mg of ibuprofen was significantly associated with lower rates of headache and nausea, as well tolerated as placebo [27]. Jirarattanaphochai and Jung [40] analyzed the effects of 400 patients treated with opioid analgesia with NSAIDs and 389 monotherapy patients with an opioid. There was no difference in the incidence of AEs between groups, consistent with the aforementioned study. In addition, several studies evaluated series of investigational formulations doses IVIB and found no renal dysfunction, gastrointestinal toxicity or bleeding events requiring blood transfusion for treatment [12, 28, 30, 36, 41]. Briefly, in line with the effectiveness and safety assessment of previous studies, IVIB 400 mg and IVIB 800 mg were safe and reliable dosages for acute postoperative pain, and also superiorly well-tolerated for patients in this protocol of the study.

### 4.3. Strengths

The strength of this study was that it is a multicenter, randomized, controlled, and prospective study. Not only were dynamic pain intensity scores considered as the important concern, but also the AUC of VAS for each time period and patient satisfaction were included in the outcome index, which was essential for the recovery of overall function. More importantly, we indicated the lack data of treatment for IVIB 400 mg on postoperative pain, compared to 800 mg and added the existing data to explain that 30 min infusion time and q6h of IVIB 400 mg and 800 mg potentially reduced complications associated with postoperative fever, such as longer hospitalization, avoidable healthcare costs, and increased morbidity for patients. In a word, it provided physicians with strategies for ibuprofen-assisted postoperative analgesia by assessing the safety and efficacy.

### 4.4. Limitations

The disadvantage was that this protocol failed to extrapolate tolerability results including relatively unhealthy patients that underwent elective surgery through broadening the inclusion/exclusion criteria. For SAEs, more detailed documentation and analysis should be done. Further, we look forward to further studies of similar design to validate the analgesic effect of intravenous ibuprofen for postoperative acute pain to add scientific validity to the conclusions, especially with IVIB 400 mg.

## 5. Conclusion

In conclusion, the results of this study suggest that IVIB 400 mg and 800 mg ibuprofen administrating postoperative pain before 30 minutes the end of surgery, with Injection every 6 hours, 8 times in total, can reduce morphine consumption, which may reduce recognized opioid-related AEs associated with postoperative analgesia, also achieved effective pain relief. Regarding the incidence of AEs associated with IVIB, gastrointestinal dysfunction, bleeding, nephrotoxicity, and cardiovascular or neurological events, there were no significant differences between the 400 mg group, the 800 mg group, and the placebo group; IVIB was well tolerated for postoperative pain treatment.

## Figures and Tables

**Table 1:** Demographic profiles and other information about patients.

Characteristics	Placebo group (*n* = 119)	IVIB 400 mg group (*n* = 115)	IVIB 800 mg group (*n* = 111)
Age (y); M ± StD	52.89 ± 10.66	52.79 ± 12.45	52.95 ± 11.13
Gender; *n* (%)			
Male	62 (52.10)	63 (54.78)	67 (60.36)
Female	57 (47.90)	52 (45.22)	44 (39.64)
Ethnicity; *n* (%)			
Han	117 (98.32)	114 (99.13)	109 (98.20)
Other	2 (1.68)	1 (0.87)	2 (1.80)
Height (cm); M ± StD	164.26 ± 7.65	164.26 ± 7.93	164.77 ± 8.26
Weight (Kg); M ± StD	62.54 ± 11.18	61.90 ± 11.60	61.50 ± 10.35
BMI (Kg/m^2^); M ± StD	23.10 ± 3.29	22.82 ± 3.18	22.56 ± 2.77
Smoking history; *n* (%)			
No	97 (81.51)	88 (76.52)	84 (75.68)
Yes	22 (18.49)	27 (23.48)	27 (24.32)
History of alcohol consumption; *n* (%)			
Never	96 (80.67)	85 (73.91)	93 (83.78)
Occasionally	11 (9.24)	17 (14.78)	5 (4.50)
Frequently	12 (10.08)	13 (11.30)	13 (11.71)
History of drug dependence; *n* (%)			
No	117 (98.32)	115 (100.00)	111 (100.00)
Yes	2 (1.68)	0 (0.00)	0 (0.00)
History of drug allergy; *n* (%)			
No	110 (92.44)	106 (92.17)	109 (98.20)
Yes	9 (7.56)	9 (7.83)	2 (1.80)
Previous surgical history; *n* (%)			
No	105 (88.24)	100 (86.96)	94 (84.68)
Yes	14 (11.76)	15 (13.04)	17 (15.32)
Another previous medical history within the past 1 y; *n* (%)			
No	59 (49.58)	60 (52.17)	61 (54.95)
Yes	60 (50.42)	55 (47.83)	50 (45.05)
Type of surgery; *n* (%)			
Abdominal surgery	58 (48.74)	60 (52.17)	56 (50.45)
Orthopaedic surgery	61 (51.26)	55 (47.83)	55 (49.55)
Classification of ASA; *n* (%)			
I	31 (26.05)	25 (21.74)	27 (24.32)
II	86 (72.27)	88 (76.52)	84 (75.68)
III	2 (1.68)	2 (1.74)	0 (0.00)
Duration of surgery (min); M ± StD	206.19 ± 95.93	215.61 ± 88.97	205.50 ± 98.09
Whether to use prophylactic antiemetic drugs; *n* (%)			
No	25 (21.01)	27 (23.48)	28 (25.23)
Yes	94 (78.99)	88 (76.52)	83 (74.77)

y: years, cm: centimeter, Kg: kilogram, min: minutes, ASA: American Society of Anesthesiologists. M ± StD: means ± standard deviation.

**Table 2:** AEs related to IVIB and morphine. (%) of patients.

Events; *n* (%)	Placebo (*n* = 119)	IVIB 400 mg (*n* = 115)	IVIB 800 mg (*n* = 111)	*P* values
Gastrointestinal dysfunction				
Naupathia	12 (10.08)	8 (6.96)	11 (9.91)	0.6487
Vomiting	10 (8.40)	4 (3.48)	8 (7.21)	0.2693
Flatulence	0 (0.00)	0 (0.00)	1 (0.90)	0.3217
Constipation	0 (0.00)	1 (0.87)	2 (1.80)	0.2129
Nervous system				
Headache	0 (0.00)	0 (0.00)	2 (1.80)	0.1029
Dizziness	1 (0.84)	1 (0.87)	3 (2.70)	0.4551
Sedation	0 (0.00)	0 (0.00)	1 (0.90)	0.3217
Renal dysfunction				
Elevated serum creatinine	0 (0.00)	1 (0.87)	0 (0.00)	0.6551
Gastrointestinal bleeding	0 (0.00)	1 (0.87)	0 (0.00)	0.6551
Cardiovascular events				
Hypotension	3 (2.52)	2 (1.74)	2 (1.80)	1
Fluctuation of blood pressure	0 (0.00)	1 (0.87)	0 (0.00)	0.6551
Allergic reactions/rash				
Skin rash	0 (0.00)	1 (0.87)	0 (0.00)	0.6551
Allergic dermatitis	2 (1.68)	0 (0.00)	0 (0.00)	0.3317
Hypersensitivity	1 (0.84)	0 (0.00)	0 (0.00)	1
Gastrointestinal bleeding	0 (0.00)	1 (0.87)	0 (0.00)	0.6551
Pruritus	0 (0.00)	1 (0.87)	0 (0.00)	0.6551
Urinary retention	2 (1.68)	1 (0.87)	2 (1.80)	0.8709
Respiratory depression	0 (0.00)	0 (0.00)	0 (0.00)	
Thrombophlebitis	0 (0.00)	0 (0.00)	0 (0.00)	

AEs: adverse events.

**Table 3:** SAEs were reported in patients.

Event	*n* (%)	Severity	Critical event types	Treatment relationship
IVIB 400 mg	2 (1.74)			
Lowing blood oxygen saturation		Moderate	Endangering life safety	Unknown
Acute anterior wall myocardial infarction		Severe	Extended hospital stay and endangering life safety	Unknown
IVIB 800 mg	3 (2.70)			
Intestinal fistula		Moderate	Other serious events	Not related
Acute cholecystitis		Severe	Extended hospital stay and other serious events	Not related
Abdominal infection		Severe	Extended hospital stay	Not related

IVIB: intravenous ibuprofen; SAEs: serious adverse events.

## Data Availability

The datasets used and/or analyzed during the current study are available from the corresponding author on reasonable request.

## Supplementary Materials

Supplementary Materials In the attachment, we have provided a total of 11 Supplemental tables, which contain the inclusion and exclusion criteria of research objects involved in this paper and the original data of data analysis, for the reference of interested friends.
