# Supplementary material for: Analgesic Efficacy of Intravenous Ibuprofen in the Treatment of Postoperative Acute Pain: A Phase III Multicenter Randomized Placebo-ControlledDouble-Blind Clinical Trial
Source: Pain Res Manag. 2023 Mar 7;2023:7768704. doi: 10.1155/2023/7768704 (PMC10014159; doi:10.1155/2023/7768704)
Supplement: Supplementary Materials — In the attachment, we have provided a total of 11 Supplemental tables, which contain the inclusion and exclusion criteria of research objects involved in this paper and the original data of data analysis, for the reference of interested friends. [file 7768704.f1.docx]

**Supplemental Instrument** **Tables**

| **Table 1. Inclusion criteria** |
| --- |
| 1. Aged between 18 and 75, male and female |
| 2. Abdominal surgery or orthopedic surgery with incisions ≥5cm is planned |
| 3. Postoperative hospitalization and patient-controlled intravenous morphine analgesia (PCIA) for at least 24 hours are required |
| 4. American Society of Anesthesiologists (ASA) physical status I-III |
| 5. The ability to reliably provide self-reports of pain |
| **Table 2. Exclusion criteria** |
| 1. Weight < 40 kg or BMI≤ 18 kg/m^2^, BMI ≥ 30 kg/m^2^ |
| 2. Hypersensitivity to NSAIDs or arginine; |
| 3. Pregnant or lactating women; |
| 4. Those on warfarin, lithium, combined angiotensin-converting enzyme inhibitors (ACEI) and diuretics; |
| 5. There are underlying bleeding factors such as hemophilia, thrombocytopenia, abnormal platelet function; |
| 6. History of asthma, poorly controlled hypertension (systolic blood pressure ≥ 140 mmHg, or diastolic blood pressure ≥ 90 mmHg); |
| 7. Patients who have used NSAIDs within 12 h |
| 8. Patients with liver dysfunction, ALT or/and AST is greater than 1.5 times the limit of the baseline; |
| 9. Patients with kidney dysfunction (creatinine is greater than 1.5 times the limit of the baseline; or On dialysis treatment within 28 days prior to surgery |
| 10. Patients who have used other analgesics, muscle relaxants or sedatives within 24h prior to surgery |
| 11. Patients who are narcotic dependent or opioid tolerant; |
| 12. Patients enrolled in other clinical studies within 3 months |
| 13. Local anesthesia, nerve blocks, epidurals, and analgesia were not allowed preoperatively/intraoperatively. |
| 14. Patients with other conditions deemed unsuitable for participation by the investigator. |

**Table 3.** **Comparison of pain intensity (VAS) at rest and with movement**

| **Time** | **IVIB 400 mg group** | | **IVIB 800 mg group** | | **Placebo group** | **P value** | |
| --- | --- | --- | --- | --- | --- | --- | --- |
| **At rest** **(P ); M±StD** |  |  |  |  |  |  |  |
| after 1 h | 21.77±22.15 | | 24.08±21.65 | | 28.59±22.07 | 0.0316 | |
| after 3 h | 23.78±18.28 | | 21.81±17.71 | | 29.85±20.62 | 0.0084 | |
| after 6 h | 20.85±17.14 | | 16.89±14.25 | | 26.81±18.34 | 0.0001 | |
| after 12 h | 16.46±14.99 | | 14.86±13.96 | | 25.23±17.31 | <0.0001 | |
| after 24 h | 14.80±14.73 | | 12.88±15.09 | | 21.60±14.74 | <0.0001 | |
| after 36 h | 12.40±12.58 | | 10.94±12.39 | | 17.80±12.97 | <0.0001 | |
| after 48 h | | 10.69±12.07 | | 9.99±11.61 | 14.76±11.85 | | 0.0009 |
| **Movement (P ); M±StD** | |  | |  |  | |  |
| after 1 h | | 29.18±22.80 | | 30.26±23.76 | 34.47±23.18 | | 0.1845 |
| after 3 h | | 23.78±18.28 | | 21.81±17.71 | 29.85±20.62 | | 0.0084 |
| after 6 h | | 25.83±18.02 | | 21.71±17.52 | 34.68±22.16 | | <0.0001 |
| after 12 h | | 22.44±17.08 | | 19.47±16.63 | 31.20±20.65 | | <0.0001 |
| after 24 h | | 21.92±17.62 | | 19.37±18.79 | 28.12±19.09 | | 0.0005 |
| after 36 h | | 12.40±12.58 | | 10.94±12.39 | 17.80±12.97 | | <0.0001 |
| after 48 h | | 16.46±14.01 | | 15.76±13.77 | 20.63±15.39 | | 0.0210 |

h: hour

| **Table 4. Comparison between groups of VAS** | | | |
| --- | --- | --- | --- |
| **Groups P** | **IVIB 400 mg group**  **vs**  **Placebo group** | **IVIB 800 mg group**  **vs**  **Placebo group** | **IVIB 400 mg group**  **vs**  **IVIB 800 mg group** |
| **At rest (P)** |  |  |  |
| after 1 h | 0.0097 | 0.0105 | 0.3078 |
| after 3 h | 0.0264 | 0.0034 | 0.4309 |
| after 6 h | 0.0066 | <0.0001 | 0.1357 |
| after 12 h | <0.0001 | <0.0001 | 0.4608 |
| after 24 h | 0.0001 | <0.0001 | 0.2325 |
| after 36 h | 0.0005 | <0.0001 | 0.3140 |
| after 48 h | 0.0030 | 0.0006 | 0.6508 |
| **Movement (P )** |  |  |  |
| after 3 h | 0.0264 | 0.0034 | 0.4309 |
| after 6 h | 0.0026 | <0.0001 | 0.0941 |
| after 12 h | 0.0013 | <0.0001 | 0.1592 |
| after 24 h | 0.0122 | 0.0001 | 0.1732 |
| after 36 h | 0.0005 | <0.0001 | 0.314 |
| after 48 h | 0.0302 | 0.0096 | 0.7008 |

h: hour

**Table 5. Comparison of** **AUCs at rest and with movement**

| **Time** | **400 mg group** | | **800 mg group** | | **Placebo group** | | **P value** | |
| --- | --- | --- | --- | --- | --- | --- | --- | --- |
| **At rest (P); M±StD** |  |  |  |  |  |  |  |  |
| after 1h-24 h | 399.70±301.30 | | 357.12±276.77 | | 585.08±344.20 | | <0.0001 | |
| after 6-24 h | 294.17±245.86 | | 261.91±232.11 | | 436.79±266.26 | | <0.0001 | |
| after 12-24 h | 188.17±164.12 | | 166.51±163.92 | | 279.74±171.60 | | <0.0001 | |
| **Movement (P); M±StD** | |  | |  | |  | |  |
| after 1-24 h | | 528.94±339.62 | | 459.99±347.49 | | 715.44±398.76 | | <0.0001 |
| after 6-24 h | | 409.31±279.19 | | 362.23±297.96 | | 557.18±324.04 | | <0.0001 |
| after 12-24 h | | 270.17±191.77 | | 236.48±208.10 | | 357.32±214.08 | | <0.0001 |

h: hour

**Table 6. Comparison between groups of AUCs at rest and with movement**

| **Groups P** | **400 mg group vs**  **Placebo group** | **800 mg group vs**  **Placebo group** | **400 mg group vs**  **800 mg group** |
| --- | --- | --- | --- |
| **At rest (P_1_ )** |  |  |  |
| after 1-24 h | <0.0001 | <0.0001 | 0.6018 |
| after 6-24 h | <0.0001 | <0.0001 | 0.6117 |
| after 12-24 h | 0.0002 | <0.0001 | 0.6089 |
| **Movement (P_2_ )** |  |  |  |
| after 1-24 h | 0.0013 | <0.0001 | 0.4000 |
| after 6-24 h | 0.0013 | <0.0001 | 0.5048 |
| after 12-24 h | 0.0063 | <0.0001 | 0.4677 |

h: hour

**Table 7. Safety assessments in the three groups**

|  | Placebo (n=119) | IVIB 400 mg (n=115) | IVIB 800 mg (n=111） | *P* value |
| --- | --- | --- | --- | --- |
| **Metabolic and nutritional diseases; n(%)** |  |  |  |  |
| Hypoproteinemia | 7(5.88%) | 22(19.13) | 9(8.11) | 0.0036* |
| Hypoalbuminemia | 2(1.68) | 4(3.48) | 3(2.70) | 0.7085 |
| Hypokalemia | 4(3.36) | 3(2.61) | 1(0.90) | 0.5454 |
| Loss of appetite | 4(3.36) | 3(2.61) | 3(2.70) | 1.0000 |
| Reduced food intake | 1(0.84) | 1(0.87) | 0(0.00) | 1.0000 |
| Hypocalcemia | 2(1.68) | 0(0.00) | 0(0.00) | 0.3317 |
|  |  |  |  |  |
| **Laboratory examination; n(%)** |  |  |  |  |
| Elevated white blood cell | 11(9.24) | 13(11.30) | 8(7.21) | 0.6032 |
| Elevated neutrophil | 3(2.52) | 3(2.61) | 0(0.00) | 0.2546 |
| Elevation of blood urea | 0(0.00) | 2(1.74) | 0(0.00) | 0.2133 |
| Elevated the monocyte count was | 1(0.84) | 0(0.00) | 0(0.00) | 1.0000 |
| Low platelet count | 0(0.00) | 0(0.00) | 2(1.80) | 0.1029 |
| Increased the percentage of Neutrophils | 0(0.00) | 0(0.00) | 1(0.90) | 0.3217 |
| Droped red blood cell count | 3(2.52) | 1(0.87) | 2(1.80) | 0.7000 |
| Hypohemoglobin | 3(2.52) | 1(0.87) | 3(2.70) | 0.6384 |
| Urine erythropoiesis | 1(0.84) | 1(0.87) | 0(0.00) | 1.0000 |
|  |  |  |  |  |
| Elevated serum creatinine | 0(0.00) | 1(0.87) | 0(0.00) | 0.6551 |
| Abnormal discharge from nasogastric tube | 0(0.00) | 0(0.00) | 1(0.90) | 0.3217 |
| Elevated alanine aminotransferase | 0(0.00) | 0(0.00) | 2(1.80) | 0.1029 |
| Urine protein | 1(0.84) | 0(0.00) | 0(0.00) | 1.0000 |
| Urine sugar | 2(1.68) | 0(0.00) | 0(0.00) | 0.3317 |
| Urine acetone bodies | 1(0.84) | 0(0.00) | 2(1.80) | 0.3200 |
| Prolonged prothrombin time | 1(0.84) | 0(0.00) | 0(0.00) | 1.0000 |
| Prolonged the activated partial thrombin time | 1(0.84) | 1(0.87) | 0(0.00) | 1.0000 |
| Prolonged blood coagulation time | 0(0.00) | 0(0.00) | 1(0.90) | 0.3217 |
| Occult blood positive | 1(0.84) | 0(0.00) | 0(0.00) | 1.0000 |
| Elevated aspartate aminotransferase | 0(0.00) | 0(0.00) | 1(0.90) | 0.3217 |
| Decreased blood albumin | 3(2.52) | 0(0.00) | 3(2.70) | 0.2245 |
| Elevated blood bilirubin | 0(0.00) | 0(0.00) | 3(2.70) | 0.0327* |
| Decreased blood potassium | 2(1.68) | 0(0.00) | 2(1.80) | 0.4729 |
| Elevated blood glucose | 0(0.00) | 0(0.00) | 1(0.90) | 0.3217 |
| Decrease in total protein | 0(0.00) | 0(0.00) | 1(0.90) | 0.3217 |
|  |  |  |  |  |
| **Basic vital signs; n(%)** |  |  |  |  |
| Elevated systolic blood pressure | 1(0.00) | 2(0.87) | 1(0.00) | 0.6551 |
| Decreased diastolic blood pressure | 0(0.00) | 1(0.87) | 0(0.00) | 0.6551 |
| Abnormal Q on electrocardiogram | 1(0.84) | 0(0.00) | 0(0.00) | 1.0000 |
| Abnormal T on electrocardiogram | 0(0.00) | 1(0.87) | 0(0.00) | 0.6551 |
| Abnormalities in heart rate | 0(0.00) | 0(0.00) | 1(0.90) | 0.3317 |
| Lowed oxyhemoglobin saturation | 0(0.00) | 1(0.87) | 0(0.00) | 0.6551 |
|  |  |  |  |  |
| **Various adverse reactions; n(%)** |  |  |  |  |
| Pyrexia | 36(30.25) | 15(13.04) | 12(10.81) | 0.0002* |
| Cheat discomfort | 2(1.68) | 1(0.87) | 1(0.90) | 1.0000 |
| Intubation-site related reactions | 0(0.00) | 0(0.00) | 1(0.90) | 0.3217 |
| Weakness | 0(0.00) | 0(0.00) | 1(0.90) | 0.3217 |
| Rigor | 1(0.84) | 0(0.00) | 1(0.90) | 0.7694 |
| Swell | 1(0.84) | 0(0.00) | 1(0.00) | 0.7694 |
| Thoracalgia | 0(0.00) | 0(0.00) | 1(0.90) | 0.3217 |
|  |  |  |  |  |
| **Gastrointestinal disorders; n(%)** |  |  |  |  |
| Naupathia | 12(10.08) | 8(6.96) | 11(9.91) | 0.6487 |
| Vomit | 10(8.40) | 4(3.48) | 8(7.21) | 0.2693 |
| Constipation | 0(0.00) | 1(0.87) | 2(1.80) | 0.2129 |
| Itchy mouth and lips | 0(0.00) | 1(0.87) | 0(0.00) | 0.6551 |
| Dry mouth | 0(0.00) | 1(0.87) | 1(0.90) | 0.5468 |
| Decreased oral sensation | 0(0.00) | 1(0.87) | 0(0.00) | 0.6551 |
| Flatulence | 0(0.00) | 0(0.00) | 1(0.90) | 0.3217 |
| Intestinal fistula | 0(0.00) | 0(0.00) | 1(0.90) | 0.3217 |
| Abdominal pain | 2(1.68) | 0(0.00) | 0(0.00) | 0.3317 |
| Distension of the abdomen | 3(2.52) | 0(0.00) | 3(2.70) | 0.2245 |
| Epigastric pain | 2(1.68) | 0(0.00) | 0(0.00) | 0.3317 |
| Dry stools | 1(0.84) | 0(0.00) | 0(0.00) | 1.0000 |
|  |  |  |  |  |
| **Blood and Lymphatic System; n(%)** |  |  |  |  |
| Anemia | 14(11.76) | 12(10.43) | 11(9.91) | 0.9063 |
| Disturbance of blood coagulation | 0(0.00) | 2(1.74) | 0(0.00) | 0.2133 |
| Hemorrhagic anemia | 0(0.00) | 1(0.87) | 0(0.00) | 0.6551 |
|  |  |  |  |  |
| **Respiratory system; n(%)** |  |  |  |  |
| Cough | 4(3.36) | 5(4.35) | 3(2.70) | 0.8755 |
| Epistaxis | 0(0.00) | 1(0.87) | 0(0.00) | 0.6551 |
| Hemoptysis | 0(0.00) | 1(0.87) | 0(0.00) | 0.6551 |
| Hiccup | 0(0.00) | 1(0.87) | 1(0.90) | 0.5468 |
| Laryngeal was unwell | 0(0.00) | 0(0.00) | 1(0.90) | 0.3217 |
| Expectoration | 4(3.36) | 0(0.00) | 3(2.70) | 0.1504 |
| Oropharyngeal discomfort | 0(0.00) | 0(0.00) | 1(0.90) | 0.3217 |
|  |  |  |  |  |
| **Circulatory system; n(%)** |  |  |  |  |
| High blood pressure | 4(3.36) | 3(2.61) | 4(3.60) | 0.9307 |
| Hypotension | 3(2.52) | 2(1.74) | 2(1.80) | 1.0000 |
| Fluctuation of blood pressure | 0(0.00) | 1(0.87) | 0(0.00) | 0.6551 |
| Acute myocardial infarction | 0(0.00) | 1(0.87) | 0(0.00) | 0.6551 |
| Tachycardia | 0(0.00) | 1(0.87) | 0(0.00) | 0.6551 |
| Atrial fibrillation | 0(0.00) | 0(0.00) | 1(0.90) | 0.3217 |
| Supraventricular external contraction | 1(0.84) | 0(0.00) | 0(0.00) | 1.0000 |
| Infranodal extrasystole | 0(0.00) | 0(0.00) | 1(0.90) | 0.3217 |
|  |  |  |  |  |
| **Surgical complication; n(%)** |  |  |  |  |
| Procedural pain | 2(1.68) | 1(0.87) | 1(0.90) | 1.0000 |
| Postoperative constipation | 0(0.00) | 1(0.87) | 0(0.00) | 0.6551 |
| Ostoperative pyrexia | 1(0.84) | 1(0.87) | 0(0.00) | 1.0000 |
| Postoperative inflammatory | 0(0.00) | 1(0.87) | 1(0.90) | 0.5468 |
| Postoperative hypertension | 1(0.84) | 0(0.00) | 1(0.90) | 0.7694 |
| Postoperative anemia | 0(0.00) | 0(0.00) | 1(0.90) | 0.3217 |
|  |  |  |  |  |
| **Infectious diseases; n(%)** |  |  |  |  |
| Abdominal infection | 0(0.00) | 1(0.87) | 2(1.80) | 0.2129 |
| Urinary tract infection | 1(0.84) | 1(0.87) | 4(3.60) | 0.2912 |
| Wound infection | 2(0.84) | 1(0.87) | 1(0.90) | 1.0000 |
| Nasopharyngitis | 0(0.00) | 0(0.00) | 1(0.90) | 0.3217 |
| Peritonitis | 0(0.00) | 0(0.00) | 1(0.90) | 0.3217 |
| Conjunctivitis | 0(0.00) | 0(0.00) | 1(0.90) | 0.3217 |
| General infection | 2(1.68) | 0(0.00) | 0(0.00) | 0.3317 |
|  |  |  |  |  |
| **Neuropsychiatric system; n(%)** |  |  |  |  |
| Hypaesthesia | 1(0.84) | 1(0.87) | 0(0.00) | 1.0000 |
| Poor sleep | 1(0.84) | 1(0.87) | 0(0.00) | 1.0000 |
| Dizzy | 1(0.84) | 1(0.87) | 3(2.70) | 0.4551 |
| sedation | 0(0.00) | 0(0.00) | 1(0.90) | 0.3217 |
| Burnout | 0(0.00) | 1(0.87) | 0(0.00) | 0.6551 |
| Insomnia | 1(0.84) | 0(0.00) | 0(0.00) | 1.0000 |
| Headaches | 0(0.00) | 0(0.00) | 2(1.80) | 0.1029 |
|  |  |  |  |  |
| **Genitourinary system dysfunction; n(%)** |  |  |  |  |
| Dysuresia | 2(1.68) | 1(0.87) | 2(1.80) | 0.8709 |
| Oliguresis | 0(0.00) | 1(0.87) | 0(0.00) | 0.6551 |
| Haematuria | 1(0.84) | 0(0.00) | 0(0.00) | 1.0000 |
| Prostatic obstruction | 0(0.00) | 0(0.00) | 1(0.90) | 0.3217 |
|  |  |  |  |  |
| **Skin and subcutaneous tissue disorders; n(%)** |  |  |  |  |
| Skin rash | 0(0.00) | 1(0.87) | 0(0.00) | 0.6551 |
| Allergic dermatitis | 2(1.68) | 0(0.00) | 0(0.00) | 0.3317 |
|  |  |  |  |  |
| **Digestive system disorder; n(%)** |  |  |  |  |
| Cholelithiasis | 1(0.84) | 0(0.00) | 0(0.00) | 1.0000 |
| Acute cholecystitis | 0(0.00) | 0(0.00) | 1(0.90) | 0.3217 |
|  |  |  |  |  |
| **Extended; n(%)** |  |  |  |  |
| Musculoskeletal pain | 1(0.84) | 0(0.00) | 0(0.00) | 1.0000 |
| Hypersensitivity | 1(0.84) | 0(0.00) | 0(0.00) | 1.0000 |

**Table 8. Comparison between groups of body temperature during the study period**

| **Time** | **400 mg group** | | **800 mg group** | | **Placebo group** | ***P* value** | |
| --- | --- | --- | --- | --- | --- | --- | --- |
| **T(℃); M±StD** | |  | |  |  | |  |
| after 1 h | | 36.49±0.32 | | 36.52±0.37 | 36.51±0.43 | | 0.8705 |
| after 3 h | | 36.64±0.40 | | 36.63±0.35 | 36.68±0.37 | | 0.6002 |
| after 6 h | | 36.69±0.41 | | 36.66±0.36 | 36.84±0.60 | | 0.0058 |
| after 12 h | | 36.73±0.42 | | 36.68±0.37 | 36.94±0.57 | | <0.0001 |
| after 24 h | | 36.81±0.38 | | 36.75±0.46 | 36.97±0.58 | | 0.0031 |
| after 36 h | | 36.78±0.44 | | 36.68±0.39 | 36.94±0.51 | | 0.0001 |
| after 48 h | | 36.83±0.44 | | 36.80±0.49 | 37.00±0.61 | | 0.0086 |

T: temperature.

**Table 9. Comparison between groups of blood routine for before and after medication administration**

| **items** | **400 mg group** | **800 mg group** | **Placebo group** | ***P* value** |
| --- | --- | --- | --- | --- |
| **RBC (×10^12^/L)****; M±StD** |  |  |  |  |
| before the first dose of medication | 4.36±0.59 | 4.44±0.55 | 4.32±0.57 | 0.2516 |
| after the last dose of  medication | 3.55±0.64 | 3.61±0.65 | 3.63±0.57 | 0.6332 |
| **HB(g/L); M±StD** |  |  |  |  |
| before the first dose of medication | 125.59±20.64 | 129.95±18.35 | 124.41±22.78 | 0.1063 |
| after the last dose of  medication | 102.56±19.19 | 106.42±21.15 | 105.15±17.89 | 0.3165 |
| **PLT(×10^9^/L); M±StD** |  |  |  |  |
| before the first dose of medication | 223.71±71.51 | 233.28±90.71 | 232.39±76.64 | 0.6056 |
| after the last dose of  medication | 182.04±70.41 | 188.68±67.20 | 193.89±65.46 | 0.4156 |
| **WBC(×10^9^/L); M±StD** |  |  |  |  |
| before the first dose of medication | 6.42±2.48 | 6.66±2.38 | 6.05±2.10 | 0.1320 |
| after the last dose of  medication | 8.95±3.01 | 8.91±3.04 | 9.40±3.24 | 0.4138 |
| **NEU(×10^9^/L); M±StD** |  |  |  |  |
| before the first dose of medication | 4.20±2.22 | 4.36±2.17 | 3.75±1.77 | 0.0699 |
| after the last dose of  medication | 7.16±2.84 | 7.00±2.99 | 7.49±2.93 | 0.4367 |
| **LYM(×10^9^/L); M±StD** |  |  |  |  |
| before the first dose of medication | 1.60±0.64 | 1.65±0.56 | 1.67±0.65 | 0.7029 |
| after the last dose of  medication | 1.09±0.54 | 1.13±0.57 | 1.17±0.63 | 0.5414 |
| **MON(×10^9^/L); M±StD** |  |  |  |  |
| before the first dose of medication | 0.43±0.20 | 0.46±0.21 | 0.42±0.19 | 0.2345 |
| after the last dose of  medication | 0.53±0.24 | 0.58±0.28 | 0.61±0.28 | 0.1178 |
| **EOS(×10^9^/L); M±StD** |  |  |  |  |
| before the first dose of medication | 0.16±0.18 | 0.15±0.13 | 0.16±0.19 | 0.6807 |
| after the last dose of  medication | 0.11±0.15 | 0.10±0.12 | 0.09±0.10 | 0.2867 |
| **BAS(×10^9^/L); M±StD** |  |  |  |  |
| before the first dose of medication | 0.02±0.02 | 0.03±0.02 | 0.03±0.02 | 0.1749 |
| after the last dose of  medication | 0.02±0.01 | 0.02±0.01 | 0.02±0.01 | P=0.7677 |

RBC: red blood cell; HB: hemoglobin; PLT: blood platelet; WBC: white blood cell; NEU: neutrophile granulocyte； LYM: lymphocyte; MON: monocyte; EOS: eosinocyte; BAS: basicyte.

**Table 10. Comparison between groups of biochemistry for before and after medication administration**

| **items** | **400 mg group** | **800 mg group** | **Placebo group** | ***P* value** |
| --- | --- | --- | --- | --- |
| **ALT(u/L); M±StD** |  |  |  |  |
| before the first dose of medication | 19.88±9.22 | 21.61±13.36 | 21.11±13.59 | 0.5488 |
| after the last dose of  medication | 17.93±16.38 | 35.48±156.66 | 16.88±9.65 | 0.2215 |
| **AST(u/L); M±StD** |  |  |  |  |
| before the first dose of medication | 21.34±6.60 | 21.68±8.64 | 20.69±7.65 | 0.6195 |
| after the last dose of  medication | 21.24±11.95 | 26.35±46.48 | 20.00±9.84 | 0.2070 |
| **ALP(u/L); M±StD** |  |  |  |  |
| before the first dose of medication | 76.77±28.06 | 81.27±35.56 | 75.72±29.91 | 0.3805 |
| after the last dose of  medication | 63.77±26.77 | 67.02±25.39 | 63.11±20.77 | 0.4653 |
| **Cr(μmol/L); M±StD** |  |  |  |  |
| before the first dose of medication | 66.36±16.97 | 68.27±15.98 | 67.79±15.71 | 0.6547 |
| after the last dose of  medication | 62.45±20.31 | 63.38±22.44 | 60.50±15.08 | 0.5257 |
| **ALB(g/L); M±StD** |  |  |  |  |
| before the first dose of medication | 40.21±4.30 | 41.24±3.67 | 41.25±4.30 | 0.0888 |
| after the last dose of  medication | 32.88±4.58 | 33.16±4.48 | 34.25±4.50 | 0.0580 |
| **BUN(mmol/L); M±StD** |  |  |  |  |
| before the first dose of medication | 5.15±1.56 | 5.18±1.86 | 5.19±1.67 | 0.9835 |
| after the last dose of  medication | 4.58±1.81 | 4.67±1.97 | 4.40±1.67 | 0.5277 |

ALT: alanine aminotransferase; AST: aspartate aminotransferase; ALP: alkaline phosphatase; Cr: creatinine; ALB: serum albumin; BUN: blood urea nitrogen.

**Table 11. Comparison between groups of coagulation for before and after medication administration**

| **items** | **400 mg group** | **800 mg group** | **Placebo group** | ***P* value** |
| --- | --- | --- | --- | --- |
| **APTT(s); M±StD** |  |  |  |  |
| before the first dose of medication | 32.00±5.86 | 31.82±5.57 | 31.70±4.95 | 0.9192 |
| after the last dose of  medication | 34.70±7.32 | 36.35±8.27 | 34.85±6.58 | 0.1959 |
| **PT(s); M±StD** |  |  |  |  |
| before the first dose of medication | 11.97±1.34 | 11.88±1.39 | 11.95±1.27 | 0.8692 |
| after the last dose of  medication | 13.08±1.98 | 12.91±1.62 | 12.80±1.24 | 0.4548 |

APTT: activated partial thromboplastin time; PT: prothrombin time.
